# Familial Alzheimer’s Disease-Related Mutations Differentially Alter Stability of Amyloid-Beta Aggregates

**DOI:** 10.1021/acs.jpclett.2c03729

**Published:** 2023-02-03

**Authors:** Nasrollah Rezaei-Ghaleh, Mehriar Amininasab, Karin Giller, Stefan Becker

**Affiliations:** † Institute of Physical Biology, Heinrich Heine University Düsseldorf, D-40225 Düsseldorf, Germany; ‡ Institute of Biological Information Processing, IBI-7: Structural Biochemistry, Forschungszentrum Jülich, D-52428 Jülich, Germany; § Department of NMR-based Structural Biology, Max Planck Institute for Multidisciplinary Sciences, D-37077 Göttingen, Germany; ∥ Department of Cell and Molecular Biology, School of Biology, College of Science, University of Tehran, 1417466191 Tehran, Iran

## Abstract

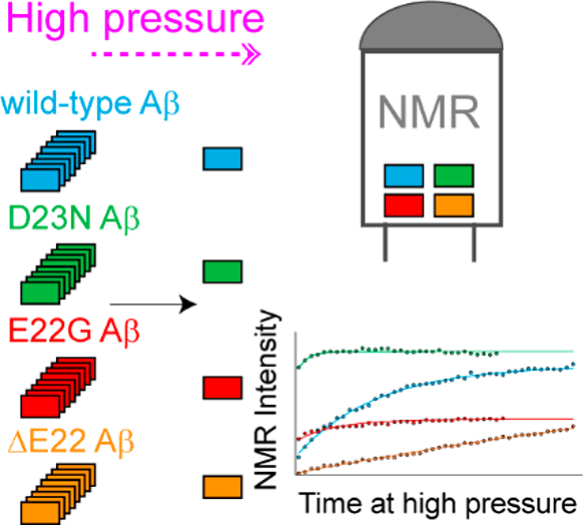

Amyloid-beta (Aβ) deposition as senile plaques is a pathological hallmark of Alzheimer’s disease (AD). AD is characterized by a large level of heterogeneity in amyloid pathology, whose molecular origin is poorly understood. Here, we employ NMR spectroscopy and MD simulation at ambient and high pressures and investigate how AD-related mutations in Aβ peptide influence the stability of Aβ aggregates. The pressure-induced monomer dissociation from Aβ aggregates monitored by NMR demonstrated that the Iowa (D23N), Arctic (E22G), and Osaka (ΔE22) mutations altered the pressure stability of Aβ40 aggregates in distinct manners. While the NMR data of monomeric Aβ40 showed only small localized effects of mutations, the MD simulation of mutated Aβ fibrils revealed their distinct susceptibility to elevated pressure. Our data propose a structural basis for the distinct stability of various Aβ fibrils and highlights “stability” as a molecular property potentially contributing to the large heterogeneity of amyloid pathology in AD.

As the most common neurodegenerative disease, Alzheimer’s disease (AD) causes 60–70% of dementia cases.^1^ Two key events in AD pathogenesis are the aggregation of a small peptide called amyloid-beta (Aβ) and the hyperphosphorylated tau protein, respectively forming extracellular senile plaques and intracellular neurofibrillary tangles in AD patients’ brains.^2^ Aβ is a 37–43 residue long peptide formed after two consecutive proteolytic cleavages of a transmembrane protein called amyloid precursor protein (APP).^3^ Most AD cases are sporadic; however, a very small fraction of AD cases follow clear and distinct inheritance patterns and arguably exhibit earlier average age at onset and more rapid progressive clinical courses.^4^ The genetic factors underlying early onset familial AD (FAD) are mainly related to mutations in genes PSEN1, PSEN2, and APP, respectively encoding proteins presenilin 1 and 2 and APP.^3^ Typically, APP mutations close to the N- or C-termini of the Aβ sequence affect total Aβ concentration or the Aβ42/Aβ40 ratio, while mutations at more internal positions are more likely to influence its aggregation propensity.^5^ Interestingly, most internal mutations of Aβ are located at residues A21 (e.g., Flemish A21G),^6^ E22 (e.g., Arctic E22G, Italian E22K, Dutch E22Q, Osaka ΔE22),^7−10^ and D23 (e.g., Iowa D23N),^11^ where modification of charge and size of amino acid side chains alter the kinetics of Aβ aggregation.^12^ A remarkable feature of FAD-related Aβ mutations is the high level of their phenotypic diversity.^3,13^ While some mutations in Aβ lead to early onset dementia and classical FAD, several other mutations, particularly those clustered at residues 21–23 are associated with cerebral amyloid angiopathy (CAA) pathology characterized by microhemorrhages and premature death.^3^

An intriguing question regarding pathological and clinical diversity of FADs is whether this phenotypic heterogeneity has its origin in the molecular properties of Aβ variants. A large body of research on the sequence dependence of in vitro Aβ aggregation demonstrates that FAD-related mutations have various effects on the rate and amount of Aβ oligomeric, protofibrillar, and fibrillar aggregation.^7,14,15^ In addition, the morphological and high-resolution structural studies of Aβ fibrils indicate a high level of fibrillar polymorphism at the micrometer and molecular scales depending on Aβ mutation type. For instance, unlike wild-type-Aβ40 fibrils with only parallel packing, the D23N-Aβ40 fibrils adopt both parallel and antiparallel packing.^16^ Another remarkable example is the ΔE22-Aβ40, which forms “cinnamon roll”-like fibrils quite distinct from wild-type-Aβ40 fibrils.^17^ In addition, the E22G mutation is shown to cause rapid formation of protofibrils in vitro and in vivo and lead to formation of several types of structurally distinct fibrils.^7,18^

Amyloid fibrils exhibit a high level of stability against mechanical and physical perturbations such as high temperature and pressure.^19,20^ Thermodynamic stability of amyloid fibrils against high hydrostatic pressure is governed by volume change upon fibril dissociation, mainly due to the presence of void volume inside the core of fibrils,^21−24^ and could therefore represent the compaction level of amyloid fibrils.^20,25^ The effect of sequence variation on the pressure stability of protein amyloid fibrils has been the subject of several recent studies.^26−28^ It has been suggested that the disease-related mutations and posttranslational modifications can modify the stability of neurodegeneration-related protein fibrils and thereby influence their pathological function, e.g., through altering their fragmentation-dependent aggregation kinetics and the prion-like spreading of aggregation pathology inside brains.^19,26^ Here, we employ high-pressure NMR and MD simulation techniques and investigate the effect of three FAD-related mutations in Aβ, i.e., E22G, D23N, and ΔE22 (Figure 1a), on pressure stability of Aβ40 fibrils.

**Figure 1:**
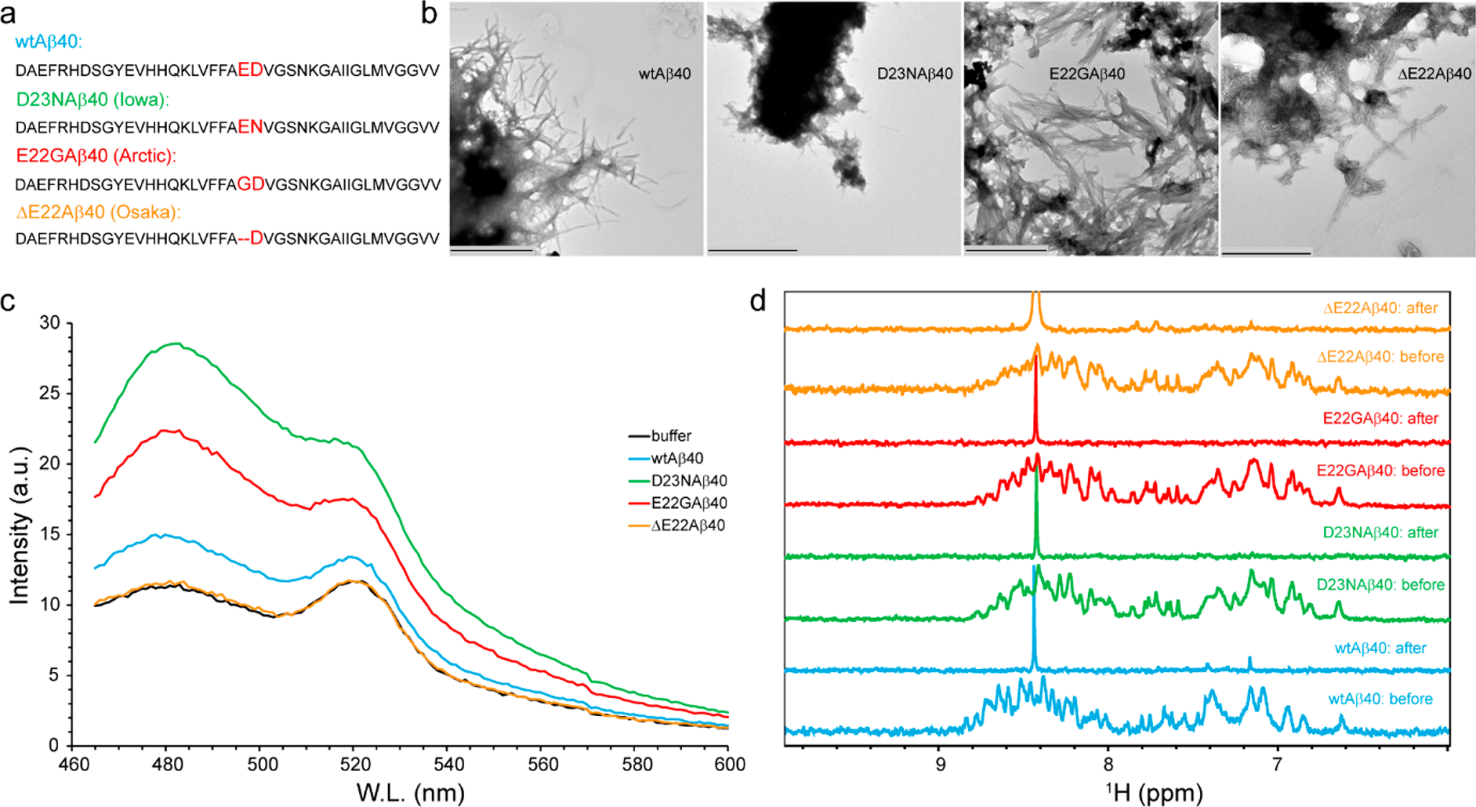
Fibrillar or nonfibrillar aggregation of Aβ40 variants. (a) Amino acid sequence of wild-type-, D23N- (Iowa), E22G- (Arctic), and ΔE22-Aβ40 (Osaka) variants, with the site of mutation highlighted. (b,c) Transmission electron microscopy (TEM) images and Thioflavin T (ThT) fluorescence emission spectra of Aβ40 samples measured after 48 h of incubation in the aggregation condition (37 °C, gentle agitation). The wild-type-, D23N-, and E22G-Aβ40 formed ThT fluorescence-enhancing fibrils, while the ΔE22-Aβ40 variant showed fibrillar aggregation without ThT fluorescence enhancement. In (b), the scale bars represent 1000 nm for the wild-type and E22G and 600 nm for the D23N- and ΔE22-Aβ40. (d) 1D ^1^H NMR spectra of Aβ40 variants measured before and after incubation in the aggregation condition. The nearly complete loss of Aβ40 signals indicate conversion of Aβ40 monomers to slowly tumbling assemblies in all the studied Aβ40 variants.

First, we established the aggregation of Aβ40 peptide variants in an aggregation-promoting condition. The transmission electron microscopic (TEM) images of Aβ samples taken after 48 h of incubation at 37 °C demonstrated abundant fibrils in the wild type-, D23N-, E22G-, and ΔE22-Aβ40 (Figure 1b). The fibrillar aggregates were predominantly found in dense networks or bundles of fibrils, as shown in Figure 1b, but individual fibrils were also observed. In addition to fibrillar aggregates, the ΔE22-Aβ40 sample contained some amorphous aggregates. Consistent with the TEM data, the wild-type-, D23N-, and E22G-Aβ40 showed significant thioflavin T (ThT) fluorescence emission. Conversely, the aggregated ΔE22-Aβ40 sample did not exhibit any considerable ThT fluorescence enhancement (Figure 1c). The lack of ThT fluorescence reactivity in ΔE22-Aβ40 fibrils is consistent with several previous reports,^14,29^ including the original report of this mutation,^10^ but contradicts another report showing an opposite fluorescence behavior of the ΔE22-Aβ40 fibrils.^30^ The Aβ40 samples were then examined through NMR measurements. The 1D ^1^H NMR spectra of Aβ40 samples measured before and after 48 h long incubation demonstrated an almost complete loss of Aβ40 signals in all the studied Aβ variants (Figure 1d). The loss of NMR signals indicate the conversion of small monomeric Aβ to large aggregate species, where the slow tumbling and consequent rapid transverse relaxation lead to severe signal broadening beyond NMR detection limit. Overall, our combined TEM, ThT fluorescence, and NMR data confirmed the fibrillar aggregation of wild-type-, D23N-, E22G-, and ΔE22-Aβ40 under the studied conditions and pointed to potential variations in the structure of their fibrils, especially in the case of ΔE22-Aβ40.

Subsequently, we investigated the stability of fibrillar aggregates of Aβ40 variants against high hydrostatic pressure. High pressure is among few perturbations (including cold temperature)^21,31^ that allow quantitative characterization of the stability of amyloid fibrils. According to the general thermodynamic principles, an increased pressure preferentially stabilizes states with lower volumes and therefore shifts the equilibrium populations toward more compact states.^21^ Since protein aggregation often leads to an imperfectly compacted structure containing water-excluded cavities, the monomer release from protein aggregates and the consequent hydration of its cavities are accompanied by volume reduction and therefore relatively favored at higher pressure levels.^22,32^ In addition, salt bridge disruptions caused by dissociation of protein aggregates could result in further volume reduction due to the electrostriction effect of separated charges on surrounding water molecules.^33^ To quantitatively determine the pressure stability of Aβ40 aggregates with dependence on mutation, we applied a high pressure level of 2000 bar (200 MPa) and monitored monomer release from Aβ40 aggregates through 1D ^1^H and 2D ^15^N,^1^H HSQC NMR measurements. At 2000 bar pressure level, a gradual increase in NMR signals reflecting the conversion of NMR-invisible Aβ aggregates to NMR-visible Aβ monomers was observed for all the four studied Aβ40 variants (Figure 2a). Interestingly, however, the rates of increase in the monomer signal were significantly different. The fastest rate of monomer release was observed for the D23N mutant, with a characteristic time of 2.6–3.5 h, followed by the E22G mutant with a characteristic time of 10.5–13.9 h. The characteristic time of monomer release from wild-type-Aβ40 was considerably longer, i.e., 24.4–26.2 h. Strikingly, the monomer release from ΔE22 mutant was too slow to be completed even after ∼4 days of pressure application, and only an apparent characteristic time of 188.9–273.1 h could be obtained. Assuming a simple model consisting of only two Aβ states, i.e., aggregated and monomeric states, and reversible dissociation of Aβ monomers from aggregates, the fitting parameters could be interpreted in terms of two first-order reaction rates, i.e., monomer dissociation (*k*_off_) and back-association (*k*_on_) rates (Figures 2b,c). The monomer dissociation rate, *k*_off_, represents the kinetic stability of aggregates at high pressure, while the ratio *k*_eq_ = *k*_off_/*k*_on_ reflects their thermodynamic stability. Based on the pressure-induced monomer release data, the estimated *k*_off_ and *k*_on_ for the wild-type-Aβ40 were respectively 6.6 ± 0.3 × 10^–6^ and 4.4 ± 0.3 × 10^–6^ s^–1^ and the *k*_eq_ was 1.5 ± 0.2, in close agreement with previous reports.^26,34^ The D23N variant had *k*_off_ of 61.6 ± 2.6 × 10^–6^ s^–1^ and *k*_on_ of 29.5 ± 1.7 × 10^–6^ s^–1^, both of them much larger than the wild-type-Aβ40 but its *k*_eq_ of 2.1 ± 0.4 was only marginally larger than the wild-type-Aβ40. These values indicate much lower kinetic stability but only slightly lower thermodynamic stability of D23N-Aβ40 aggregates compared to wild-type-Aβ40 aggregates. In comparison, the E22G variant showed a similar *k*_off_ of 6.9 ± 1.9 × 10^–6^ s^–1^, but its *k*_on_ of 15.8 ± 1.9 × 10^–6^ s^–1^ was significantly larger than the wild-type-Aβ40. Consequently, the E22G had a *k*_eq_ of 0.44 ± 0.49, significantly smaller than the wild-type-Aβ40, indicating the higher thermodynamic stability of E22G-Aβ40 aggregates. The monomer dissociation and back-association rates for ΔE22 variant were much smaller (*k*_off_ = 1.0 ± 0.0 × 10^–6^ s^–1^; *k*_on_ = 0.2 ± 0.1 × 10^–6^ s^–1^) and the equilibrium constant was larger (*k*_eq_ = 3.8 ± 0.5), suggesting their higher kinetic but lower thermodynamic stability. It should however be noted that, due to incomplete monomer release, the *k*_eq_ value of ΔE22-Aβ40 should be taken with caution and is not strictly reliable. It is also worth noting that the equilibrium and rate constants obtained here at high pressure and low temperatures are not directly comparable with thermodynamic and kinetic parameters (e.g., critical concentration for aggregation, lag times, nucleation and elongation rates) of Aβ aggregation often determined at ambient pressure and higher temperatures. Overall, the pressure-induced monomer release data demonstrate the differential impact of these FAD-related mutations on the kinetic and thermodynamic stability of Aβ aggregates.

**Figure 2:**
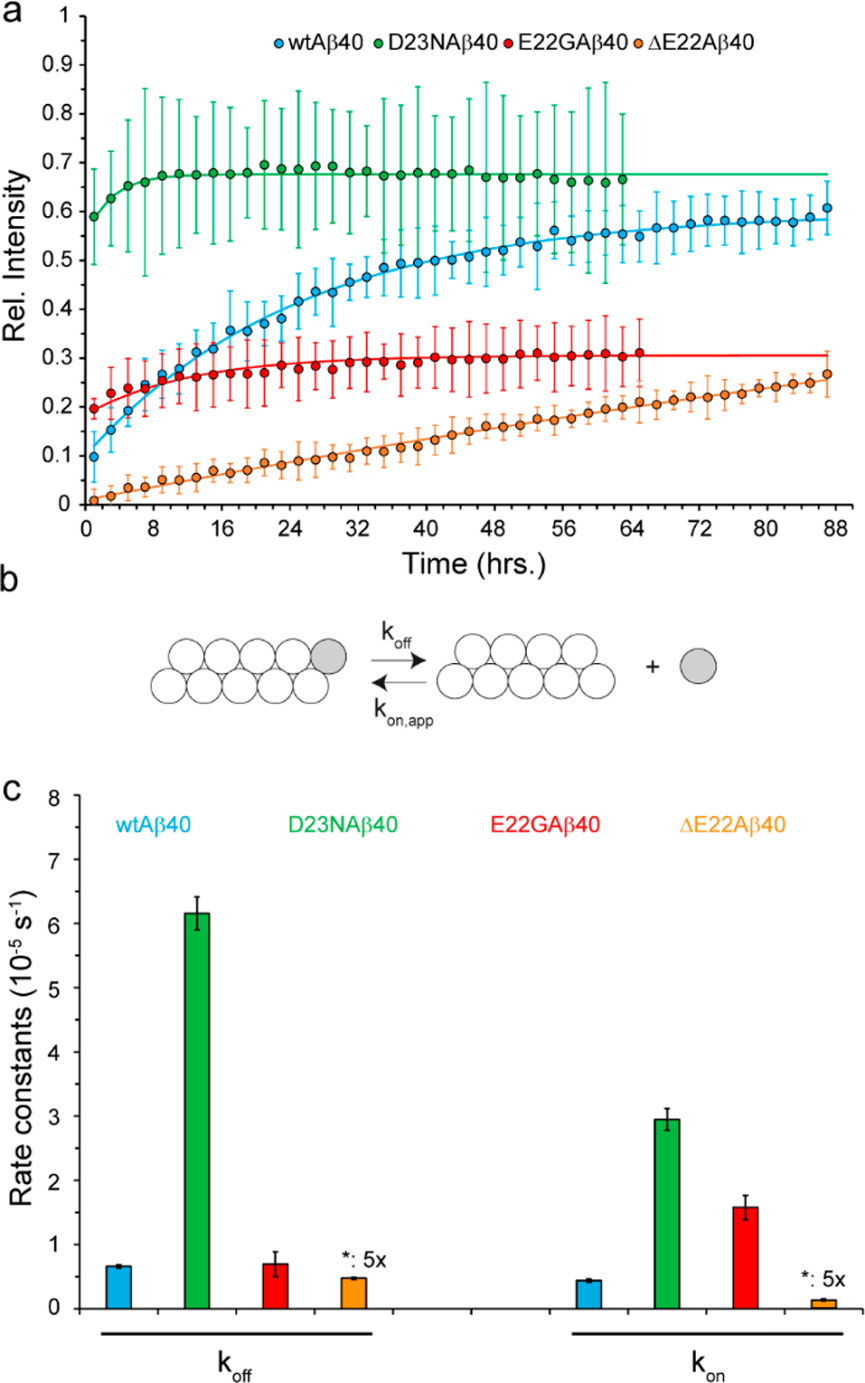
Stability of Aβ40 aggregates against high pressure. (a) Pressure-induced monomer release from Aβ40 aggregates, as followed by real-time NMR experiments at 2000 bar, 278 K, through average (±SD) peak intensities. (b) A simple kinetic model of Aβ40 disaggregation, involving only two states (aggregate state, monomer state) and two rates (dissociation, *k*_off_, and back-association, *k*_on_). (c) Rate constants obtained from the analysis of monomer release kinetic data shown in (a), according to the simple model shown in (b). The D23N-Aβ40 variant showed much larger *k*_off_ and *k*_on_ rates than the wild-type variant, while the ΔE22-Aβ40 variants showed smaller *k*_off_ and *k*_on_ rates. See the text for further details and interpretations. For the sake of visibility, the rate constants of ΔE22-Aβ40 are multiplied by a scaling factor of 5. The error bars represent fitting errors.

The relative stability of Aβ monomers and aggregates is determined by the structural and dynamical properties of Aβ at the level of monomeric and aggregate states. The monomeric Aβ is known to be largely unstructured in solution and to populate a heterogeneous conformational ensemble with a slight propensity to adopt transient secondary structures.^35,36^ NMR chemical shifts of protein backbone nuclei are sensitive probes of local conformation and can be used to predict the probability of various secondary structural motifs in proteins.^37,38^ To investigate the effect of FAD mutations on the structural properties of Aβ monomer ensembles, we measured a set of backbone chemical shifts (CO, Cα, N, HN, Hα plus Cβ) in wild-type-, D23N-, E22G-, and ΔE22-Aβ40. As shown in Figure 3a (and Supplementary Figure S1), the mutation-induced perturbations in backbone amide chemical shifts were largely localized around the site of mutation, i.e., residues F20-G25, although smaller yet significant perturbations were also observed further proximal at residues H14-L17. Interestingly, the HN (and N) of G22 in E22G-Aβ40 exhibited remarkable upfield chemical shift (Supplementary Figures S1 and S2), which is likely due to the ring current effect of proximal phenylalanine residues (F20 or F19), as suggested previously.^39^ The backbone chemical shift-based prediction of secondary structures indicated the propensity of wild-type-Aβ40 to adopt a loop-β-loop-β structure, in which the two β-strands approximately extend over residues L17-F20 and I31-V36 (Figure 3b and Supplementary Figure S3). Upon mutations, the secondary structural propensity of Aβ40 is largely preserved, however, the local structure at the site of mutation is altered with dependence on mutation type. While residues 22–25 tend to form higher/lower level of β-strands/loops after the D23N mutation, an opposite structural tendency is observed upon ΔE22 and particularly E22G mutations.

**Figure 3:**
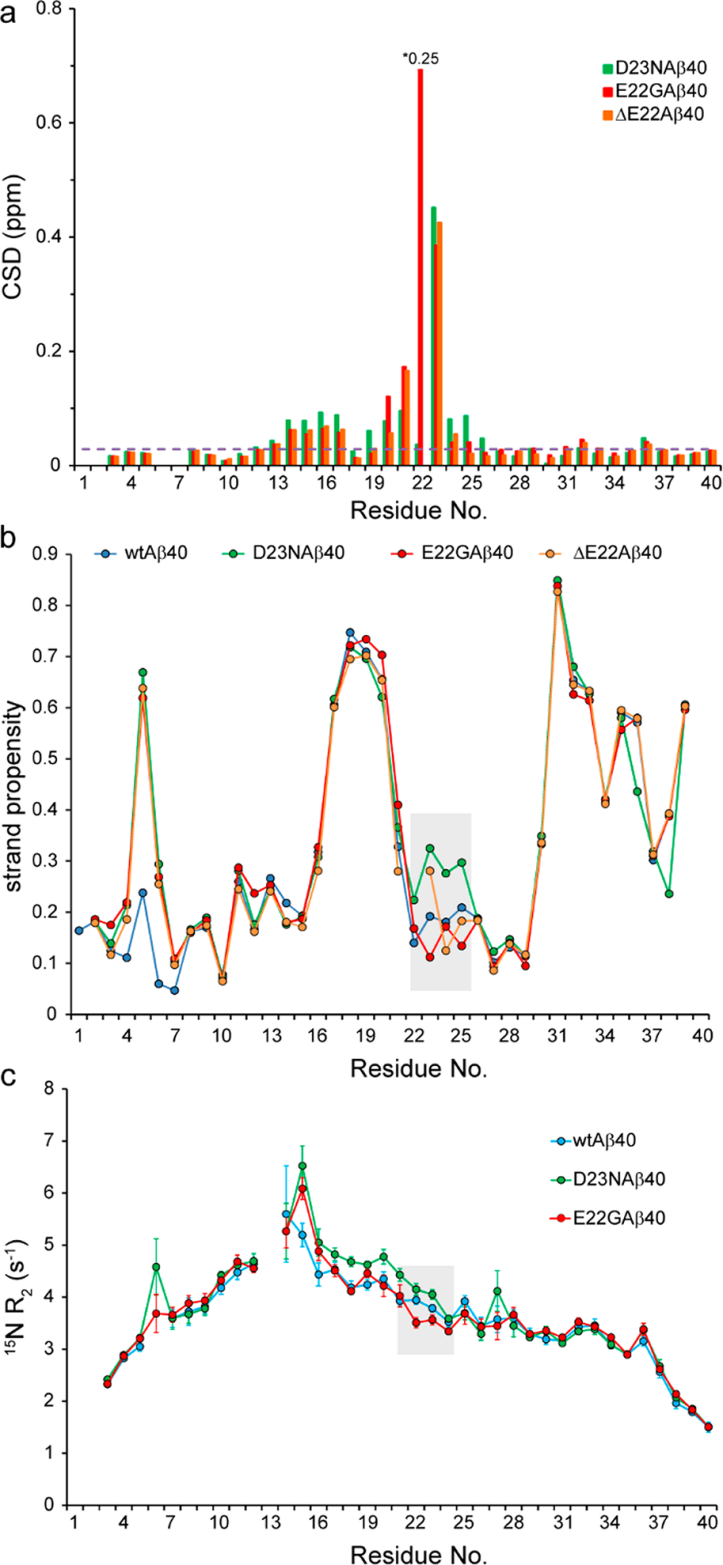
Effect of FAD mutations on the structure and dynamics of Aβ40 variants. (a) Combined ^15^N and ^1^H chemical shift deviation (CSD) of D23N-, E22G-, and ΔE22-Aβ40 peptides with respect to the wild-type-Aβ40, showing significant chemical shift differences around the respective mutation sites and to a lower extent around residues H14-L17. The dashed line represents the noise level in chemical shift values. For the sake of visibility, the CSD value of residue 22 in the E22G-Aβ40 peptide has been scaled down by a factor of 4. (b) The strand propensity of wild-type-, D23N-, E22G-, and ΔE22-Aβ40, calculated on the basis of their backbone (CO, Cα, N, HN, Hα) plus Cβ chemical shifts. The shaded area shows how the respective mutations alter the strand propensity around the site of mutation (see also Supplementary Figure S3). (c) Residue-specific ^15^N transverse relaxation (*R*_2_) rates of Aβ40 variants measured at 278 K. Note the larger *R*_2_ of D23N-Aβ40 and smaller *R*_2_ of E22G-Aβ40 around the mutation site (shaded area), indicating respectively their relative rigidity and flexibility when compared with the wild-type-Aβ40. The relative rigidity of the D23N-Aβ40 variant extends further proximally to Q15-K16.

As an intrinsically disordered peptide/protein (IDP), Aβ backbone dynamics contain multiple modes of motions spanning over a broad range of length and time scales.^40,41^ To investigate how FAD mutations influence backbone dynamics of Aβ40 monomers, we measured the ^15^N *R*_2_ relaxation rates of Aβ40 variants at three temperatures of 278, 283, and 288 K. The ^15^N *R*_2_ rates are particularly sensitive to slow reorientational dynamics of peptides and conformational exchange processes and therefore report peptide backbone motions at around nanoseconds and micro- to milliseconds time scales. In general, the values and sequence profiles of the ^15^N *R*_2_ and their temperature dependence were highly similar in different Aβ variants. However, in the region of mutation, residues 22 and 23 showed slightly larger *R*_2_ rates (less flexibility) in D23N and smaller *R*_2_ rates (more flexibility) in E22G, when compared to the wild-type-Aβ40 (Figure 3c and Supplementary Figure S4). These local changes of Aβ dynamics were in line with the secondary structural propensity changes: the partially rigidified residues of D23N exhibit an increased propensity for strand formation, while the partially mobilized residues of E22G show a decreased strand propensity. Therefore, except for the small structural and dynamical changes largely restricted to the vicinity of mutation sites, the difference in the structural dynamics of Aβ variants at the monomeric level does not seem to be sufficiently large to account for the pronounced differences observed in the stability of Aβ aggregates.

To investigate whether distinct pressure stability of Aβ40 variants originated from the structural dynamics of their fibrils, we performed 200 ns-long MD simulation of Aβ fibrils at ambient and high pressures. The MD simulations were performed using as initial structures the 2m4j model for the wild-type-Aβ40 fibrils,^42^ the 2mpz model for the D23N-Aβ40 fibrils,^43^ and the 2mvx model for the ΔE22-Aβ40 fibrils.^17^ No structural model was found for E22G-Aβ40 fibrils. The structure of wild-type- and D23N-Aβ40 fibrils comprises three cross-β units, with a 3-fold symmetry axis about the long fibril axis, while the ΔE22-Aβ40 fibrils consist of two cross-β units with 2-fold symmetry (Supplementary Figure S5). The wild-type- and ΔE22-Aβ40 fibrils exhibit a predominantly negative electrostatic surface potential, while the D23N-Aβ40 fibrils have a largely positive surface potential (Figure 4a). In the wild-type- and D23N-Aβ40 fibrils, the total interchain binding energy (consisted of van der Waals and electrostatic interactions and (de)solvation of polar and nonpolar groups) becomes negative only at sufficiently large internal dielectric constants (ε of 6 for the wild-type-Aβ40 and 4 for D23N-Aβ40 fibrils) when positive energy term associated with repulsive electrostatic interaction is significantly scaled down (Supplementary Figure S6). In contrast, the interchain electrostatic interaction of ΔE22-Aβ40 fibrils is attractive (negative energy) and the total binding energy is negative across the studied ε range of 2–7 (Supplementary Figure S6). The packing efficiency of wild-type-, D23N-, and ΔE22-Aβ40 fibrils, calculated as the ratio of van der Waals volume to Voronoi volume, is 0.682, 0.670, and 0.666, respectively, indicating slightly higher packing of wild-type-Aβ40 fibrils than the two mutated fibrils.

**Figure 4:**
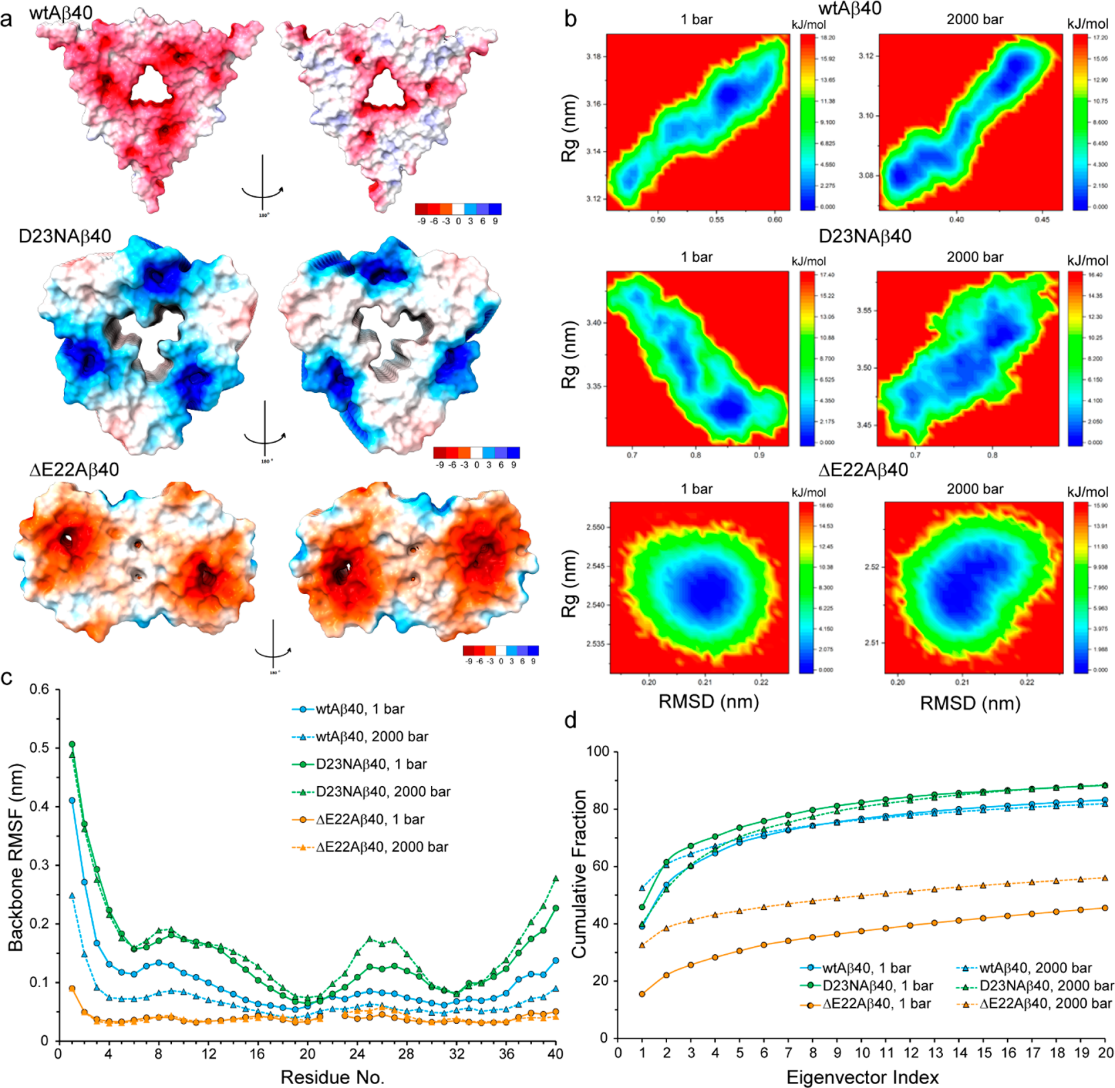
MD simulation of wild-type-, D23N-, and ΔE22-Aβ40 fibrils at ambient and high pressure levels. (a) The surface electric potential of wild-type-, D23N-, and ΔE22-Aβ40 fibrils, showing predominantly negative potential for the wild-type and ΔE22 but positive potential for D23N-Aβ40 fibrils. (b) Free energy landscape of different Aβ40 fibrils, plotted as the radius of gyration (*R*_g_) versus root-mean-square of deviation (RMSD) of backbone atoms positions, evaluated at two pressure levels of 1 and 2000 bar. (c) The root-mean-square of fluctuations (RMSF) of backbone atoms for different Aβ40 fibrils, evaluated at two pressure levels of 1 and 2000 bar. (d) Cumulative contribution of the 20 largest modes of motion, as obtained from the principal component analysis of backbone motions in the MD trajectories of different Aβ40 fibrils at two pressure levels of 1 and 2000 bar.

Upon pressure increase to 2000 bar, the radius of gyration (*R*_g_) of the wild-type-Aβ40 fibril decreased to 0.98 ± 0.01 of its value at 1 bar. In comparison, the ΔE22-Aβ40 fibril exhibited a ratio of 0.99 ± 0.00 indicating slight compaction similar to wild-type-Aβ40 fibrils, while the D23N-Aβ40 fibril showed a distinctive ratio of 1.03 ± 0.02, representing an opposite behavior, i.e. slight expansion. The *R*_g_ vs RMSD landscape of wild-type-Aβ40 fibrils at 1 bar revealed two separate low-free energy regions, one of them a small region of relatively compact structures (Figure 4b). Upon pressure increase to 2000 bar, this region was further stabilized and the free energy barrier between the two regions was significantly reduced. The ΔE22-Aβ40 fibril showed a single low-free energy region, which upon pressure increase from 1 to 2000 bar, was shifted toward more compact structures (Figure 4b). In contrast, the energy landscape of D23N-Aβ40 fibrils at 1 bar showed a small low-free energy region of extended structures along with several other stable regions populated by relatively compact structures (Figure 4b). Interestingly, the pressure rise to 2000 bar led to further stabilization of the extended region and reduction in the energy barrier between the extended and compact regions.

Subsequently, we investigated the volumetric properties of Aβ40 fibrils through calculating their packing efficiency at 1 and 2000 bar. The packing efficiency of wild-type- and D23N-Aβ40 fibrils showed no significant alteration after pressure elevation in the MD simulation: the packing efficiency of wild-type-Aβ40 fibril was 0.679 ± 0.014 at 1 bar and 0.671 ± 0.013 at 2000 bar, whereas the corresponding values for the D23N-Aβ40 fibril were 0.664 ± 0.010 at 1 bar and 0.664 ± 0.011 at 2000 bar. On the other hand, the packing efficiency of the ΔE22-Aβ40 fibril exhibited a considerable albeit small increase from 0.669 ± 0.008 at 1 bar to 0.672 ± 0.008 at 2000 bar. The higher packing efficiency of the ΔE22-Aβ40 fibril at 2000 bar suggested its larger compressibility than the wild-type- and D23N-Aβ40 fibrils, in which the packing efficiency remained nearly constant despite pressure rise.

Next, to examine how the electrostriction effect by separated charges might affect pressure-dependent volume changes in the studied systems, the separation distance between the oppositely charged groups of Aβ40 fibrils were determined at two pressure levels of 1 and 2000 bar. To this end, we calculated distances between a number of selected charge pairs, including the positively charged N-terminal amino group, R5 and K28 side chains in one hand and D1, E3, D7, E11, E22, and D23 side chains in the other hand, whether they were located inside the same peptide chain or in different chains of the same or different layers. As reported in Supplementary Table S1, the effect of pressure elevation on charge separation distances was rather heterogeneous. However, when the ratios between distances obtained at 2000 and 1 bar were averaged over all selected pair groups, a trend could be observed: the average distance ratio (2000 bar: 1 bar) was 0.93 ± 0.12 for the wild-type-Aβ40, 1.01 ± 0.03 for the D23N-Aβ40, and 1.03 ± 0.06 for the ΔE22-Aβ40 fibrils. These data provide further support for different pressure responses of the studied Aβ fibrils. While it is not straightforward to interpret these data in terms of the electrostriction effect contribution to the compressibility of Aβ40 fibrils, one might argue that the larger (average) charge separation distances of ΔE22-Aβ40 fibril at 2000 bar may enhance the electrostriction effect and contribute to its larger compressibility.

Subsequently, we examined the effect of pressure rise on the local and long-range dynamics of Aβ40 fibrils. The backbone root-mean-square-fluctuations (RMSF) of wild-type- and ΔE22-Aβ40 fibrils indicated their relative rigidity at 1 bar, which in the case of wild-type-Aβ40 was further enhanced at high pressure (Figure 4c). Conversely, the D23N-Aβ40 fibril enjoyed relatively high backbone dynamics, which became more pronounced at higher pressure especially at residues 22–28 (Figure 4c). Since the backbone RMSFs were calculated after removal of global rotational and translational motion of the whole molecules, the motions represented by them are expected to be largely dominated by fast motions occurring on pico-to-nanosecond time scales. Furthermore, the principal component analysis of peptide motions provided information on the concerted structural dynamics in Aβ fibrils with dependence on pressure (Figure 4d). The eigenvalues are the average square displacement along the corresponding eigenvectors and represent the amplitude of a concerted motional mode in the studied systems. In wild-type- and ΔE22-Aβ40 fibrils, the pressure rise to 2000 bar increased the fraction of the top three eigenvalues, especially in ΔE22-Aβ40 fibrils where the cumulative fraction of the top three eigenvalues exhibited ca. 2-fold increase (Figure 4d). An opposite effect was observed in the D23N-Aβ40 fibril, where the pressure rise to 2000 bar caused a decrease in the cumulative fraction of the top five eigenvalues. These data suggest a pressure-dependent reduction in the amount of concerted dynamics in D23N-Aβ40 fibrils, while the concerted dynamics of the wild-type- and particularly ΔE22-Aβ40 fibrils are enhanced at high pressure. In line with their distinct dynamical responses to high pressure, the Aβ40 fibrils were differently affected in terms of intermolecular hydrogen bonds. While upon pressure elevation to 2000 bar the average number of intermolecular H-bonds rose by ∼7 and 6%, respectively in the wild-type- and ΔE22-Aβ40 fibrils, the D23N-Aβ40 fibril exhibited an opposite behavior by ∼8% drop in the number of intermolecular H-bonds. Overall, the MD data clearly demonstrated the distinctive behavior of Aβ40 fibrils, in particular the D23N-Aβ40 fibril, in response to pressure rise and suggested that their different thermodynamic and kinetic stability against high pressure is predominantly rooted in their fibrillar, rather than monomeric, states.

Several aspects of Aβ aggregation are already known to be affected by FAD mutations within Aβ sequence. These mutations can change the rate and amount of Aβ aggregation, alter the morphological and structural properties of Aβ fibrils, and modulate their susceptibility to the proteolytic degradation machinery.^7,14,15^ Our data reveal that the FAD mutations within the Aβ sequence can also influence another molecular property of Aβ fibrils, which is their stability against pressure-induced monomer dissociation. The effect of sequence modifications on the stability of amyloid fibrils has already been shown,^44^ e.g., in the case of Ser-8-phosphorylated Aβ,^26^ in which it was suggested that the stability-altering modification contributes to the progressive course of AD pathology and the conversion from preclinical to symptomatic AD.^45^ Recently, immunohistochemical and amyloid PET imaging studies have shown different regional and temporal patterns of amyloid accumulation with dependence on the mutation type in mouse models and FAD cases.^46^ Our data demonstrate that the Aβ sequence-modifying FAD mutations alter the thermodynamic and kinetic stability of Aβ40 fibrils in different ways (Figure 2): the D23N mutation decreases the thermodynamic and particularly kinetic stability, the E22G mutation increases the thermodynamic but not the kinetic stability, and the ΔE22 mutation increases the kinetic stability of Aβ40 fibrils. Accordingly, we propose that the stability alteration of Aβ fibrils caused by FAD mutations is capable of modulating the spatiotemporal spreading patterns of Aβ aggregation pathology in human AD brains and potentially contributes to the large level of phenotypic diversity among them.^13^

Thermodynamic stability of protein aggregates against pressure-induced monomer release is determined by the change in partial molar volume, Δ*V*_A→m_, from the aggregate (A) to the monomeric (m) state. If the compressibilities of A and m states are different, then Δ*V*_A→m_ will become pressure-dependent.^21,24^ For example, with everything else remaining the same, an increase in the compressibility of A state will lead to a reduction in Δ*V*_A→m_ at higher pressure levels. The main structural determinants of Δ*V*_A→m_ are (*i*) the presence of water-excluded cavities in the aggregate state and (*ii*) the difference in solvent density within the hydration layer of the two states.^24^ If protein aggregates contain water-excluded cavities, as is frequently the case for protein amyloid fibrils,^20,47^ monomer dissociation will lead to the exposure and filling of cavities by surrounding water and therefore reduce the system volume (Δ*V*_A→m_ < 0). Consequently, protein disaggregation will be favored at higher pressure levels. In addition, if protein disaggregation involves disruption of salt bridges, the electrostriction of water molecules by separated charges will reduce the system volume, hence providing further stabilization energy at higher pressure levels for the disaggregated state.^48^ As shown by the NMR chemical shift and ^15^N relaxation data (Figure 3 and Supplementary Figures S3 and S4) and in line with a previous high-pressure NMR study,^39^ the structure and backbone dynamics of monomeric Aβ40 remain largely intact upon FAD mutations, except in the vicinity of mutation sites. While the D23N mutation induced a local rigid strand conformation, the E22G mutation exhibited an opposite effect and favored a local mobile coil-like conformation. In sharp contrast with the small localized effects of FAD mutations in the intrinsically disordered monomeric state of Aβ, the solid-state NMR-based structural models of Aβ fibrils demonstrate significant structural differences among mutated Aβ40 fibrils.^17,18,43^ Equally, the MD data presented here reveal significant differences in the backbone dynamics of mutated Aβ40 fibrils at ambient and high pressure levels (Figure 4). Besides, the pressure-dependent changes in packing efficiency and charge separation distances in the studied Aβ40 fibrils suggest their different compressibilities. Furthermore, the interchain binding energy calculation with dependence on dielectric constant supports a relatively wet interchain interface for the D23N and particularly wild-type-Aβ40 fibrils (larger ε values) compared to the ΔE22-Aβ40 fibril (Supplementary Figure S6). This is consistent with previous 2D IR spectroscopy data showing the presence of trapped water molecules in wild-type-Aβ fibrils^49^ and may influence the degree of compaction in these fibrils. Our data indicate that the difference in stability of various Aβ fibrils with respect to their monomeric state originates mainly in the fibrillar, instead of the monomeric, state. Remarkably, the less stable D23N-Aβ40 fibril shows features such as pressure-dependent increase in radius of gyration and local dynamics, pressure-dependent decrease in the number of intermolecular hydrogen bonds and concerted long-range dynamics, and low compressibility. Conversely, the pronounced features of the more stable ΔE22-Aβ40 fibril were a relatively dry interchain interface and pressure-dependent increases in packing efficiency and (average) charge separation distances, hence its larger compressibility. It is, however, notable that the relevance of our MD results relies on the assumption that the structural models used in the MD simulations represent the predominant structure of fibrils generated in vitro or in vivo, which is difficult to assess in the context of the present study. Besides, various degrees of structural polymorphism potentially existing in aggregated samples of Aβ40 variants, for example, due to secondary nucleation at relatively high concentrations of Aβ used in this study and agitation-induced fragmentation, could partially contribute to the observed differences in their pressure-induced monomer dissociation behavior.

In summary, the pressure stability of wild-type-Aβ40 and FAD-related D23N-, E22G-, and ΔE22-Aβ40 fibrils was measured through a high pressure real-time NMR method. The pressure-induced monomer release data revealed the various effects of FAD mutations on the stability of Aβ40 fibrils. Most notably, the D23N mutation reduced the thermodynamic and kinetic stability of Aβ40 fibrils, while the E22G or ΔE22 mutations increased the thermodynamic or kinetic stability of Aβ40 fibrils, respectively. The combined NMR and ambient and high-pressure MD simulation data suggested that the altered stability of Aβ40 fibrils with respect to Aβ40 monomers predominantly originated in the fibrillar state. Our results provide experimental support and mechanistic insight on the stability-modulating effects of FAD mutations and highlight fibrillar “stability” as a molecular property potentially contributing to the large level of clinical and pathological heterogeneity in FAD and other neurodegenerative diseases.

## Supporting Information Available

Details on materials, aggregation, and NMR experiments, analysis of high-pressure NMR data, MD simulation, Figures S1–S6 of HSQC spectra, loop propensity, transverse relaxation rates, structures, binding energy per strand, and Table S1 of distance between oppositely charged groups (PDF)
