# Supplementary material for: Familial Alzheimer’s Disease-Related Mutations Differentially Alter Stability of Amyloid-Beta Aggregates
Source: J Phys Chem Lett. 2023 Feb 3;14(6):1427–35. doi: 10.1021/acs.jpclett.2c03729 (PMC9940190; doi:10.1021/acs.jpclett.2c03729)
Supplement: Supplementary file 1 — jz2c03729_si_001.pdf [file jz2c03729_si_001.pdf]

Familial Alzheimer's Disease-Related Mutations Differentially Alter Stability of Amyloid-Beta Aggregates.

Nasrollah Rezaei-Ghaleh,[a,b,c] Mehriar Amininasab,[d] Karin Giller,[c] and Stefan Becker [c]*

[a]Institute of Physical Biology, Heinrich Heine University Düsseldorf, D-40225 Düsseldorf, Germany

[b]Institute of Biological Information Processing, IBI-7: Structural Biochemistry, Forschungszentrum Jülich, D-52428 Jülich, Germany

[c]Department of NMR-based Structural Biology, Max Planck Institute for Multidisciplinary Sciences, D-37077 Göttingen, Germany

[d]Department of Cell and Molecular Biology, School of Biology, College of Science, University of Tehran, 1417466191 Tehran, Iran

Supplementary Methods

Materials

Uniformly ^{15}N,^{13}C-labelled recombinant Aβ1-40 (Aβ40) was produced as follows. Aβ40 was expressed as a fusion protein using a modified pET28a vector (Novagen). Protein expression was performed in E. coli strain BL21(DE3) at 37 °C in minimal medium with ^{15}N-ammonium chloride as nitrogen source and $^{13}C_6$-β-D-glucose as carbon source. After purification on a nickel column (Macherey-Nagel) the fusion protein was digested overnight on ice with TEV protease. Final purification was performed on a C4 reversed phase Vydac HPLC column. The released Aβ (1-40) peptide eluted in a linear (0-100%) acetonitrile gradient from this column as a single peak. The purified peptide was lyophilized before use. The constructs for the Iowa (D23N), Arctic (E22G) and Osaka (ΔE22) variants of the Aβ40 peptide were generated by PCR mutagenesis. The mutant proteins were expressed and purified using the protocol established for wild-type Aβ40. To disaggregate the pre-formed aggregates, the lyophilized Aβ40 peptide powders were dissolved in 20 mM NaOH at a concentration of 2 mg/mL (~ 460 μM), as described before. The monomerized Aβ40 solutions were then aliquoted, flash frozen in liquid nitrogen and stored at -80 °C until use.

Aggregation experiments

The monomerized samples of wild-type, D23N, E22G and ΔE22 Aβ40 were prepared at 0.4 mg/mL (~ 92 μM) concentration and pH 7.4, buffered with 25 mM HEPES containing 50 mM sodium chloride. The Aβ40 samples were incubated at 37 °C for 48 hours under gentle stirring by tiny magnetic rods. At the end of incubation time, the formation of Aβ40 aggregates was confirmed by electron microscopy and Thioflavin T (ThT) fluorescence assay. For electron microscopy, the

Aβ40 samples were diluted with the above-mentioned buffer, deposited onto carbon-coated copper mesh grids and negatively stained with 2% (w/v) uranyl acetate. After washing out the excess stain and drying the sample grids under air, the samples were visualized using a Philips CM 120 BioTwin transmission electron microscope (Philips Inc.). The ThT binding to Aβ aggregates was quantified through measuring fluorescence emission at 482 nm with excitation at 446 nm using a Cary Eclipse fluorimeter.

NMR experiments

NMR experiments were recorded at proton Larmor frequencies of 599.9 and 701.15 MHz using Bruker (Karlsruhe, Germany) spectrometers equipped with cryogenic probes. Before NMR experiments, temperature calibration was performed using a standardized thermocouple, and temperature control to ±0.05 K during NMR experiments was achieved using the Bruker VT unit. Unless specified otherwise, the NMR samples contained 0.4 mg.mL-1 of uniformly-^{15}N,^{13}C-labeled Aβ40 peptides (ca. 92 μM) dissolved in 25 mM HEPES, 50 mM NaCl, pH 7.4. The NMR samples contained 10% (v/v) D2O for spectrometer frequency locking, and around 0.2 mM DSS for chemical shift referencing (0.000 ppm). Peak assignments for wild-type and D23N-, E22G- and ΔE22-Aβ40 variants were obtained through conventional 2D ^{15}N,^{1}H- and ^{13}C,^{1}H-HSQC and triple resonance 3D HNCO, HNCA and CBCA(CO)NH experiments at 278 K. Peak assignments at other temperatures were obtained through small temperature increments and transfer of peak assignments (Figure S2).

^{15}N transverse relaxation (R_2) rates of wild-type, D23N- and E22G-Aβ40 variants were measured at proton Larmor frequency of 701.15 MHz at specified temperatures. Due to poor signal to noise ratio, no ^{15}N R_2 measurement could be done for the ΔE22-Aβ40 variant. The ^{15}N R_2 rates were

measured through a CPMG-based NMR pulse sequence at a CPMG frequency, ν_{CPMG}, of 1000 Hz, i.e. τ_{cp} = 1 ms, using 5.56 kHz ^{15}N π-pulses applied at the middle of τ-π-τ blocks for refocusing ^{15}N chemical shift and ^{15}N,^{1}H scalar coupling evolution. Nine relaxation delays ranging from 12 to 400 ms were used and one relaxation delay was measured twice in order to check for the stability of experiment and evaluate the uncertainty of obtained rates. The pulse sequence contained a heat compensation element at the beginning of recycle delay d_1. A total recycle delay (d_1 + acq.) of 3 s was used. The residue-specific ^{15}N R_2 rates were determined through fitting the peak intensity vs relaxation delay data to single-exponential decay functions. The corresponding errors were estimated via 50 Monte-Carlo (MC) simulation runs using fitting residuals as random noise.

High-pressure NMR experiments

Pressure-induced monomer release from Aβ aggregates were monitored using a 701.13 MHz Bruker spectrometer equipped with a cryogenic probe connected to a high-pressure device (Daedalus Innovations LLC, PA). After 48-hour incubation of Aβ40 variants in the aggregation-promoting condition (0.4 mg/mL peptide concentration, pH 7.4 buffered with 25 mM HEPES containing 50 mM sodium chloride, 37 °C, gentle stirring), the aggregated Aβ40 samples were transferred to a ceramic high-pressure NMR tube and subjected to ambient or high hydrostatic pressures. The 1D ^{1}H and 2D ^{15}N,^{1}H HSQC spectra at 278 K and ambient pressure confirmed the nearly 100% loss of Aβ monomer signal intensity due to aggregation. The pressure was then rapidly elevated to 2000 bar and the pressure-induced monomer release was monitored through consecutive 1D ^{1}H and 2D ^{15}N,^{1}H HSQC experiments. Due to the need for pressure equilibration and setting-up the NMR experiments at higher pressure, there was ~ 0.5-1 hour delay between pressure elevation and the first NMR experiment at higher pressure. Consequently, as shown in

Fig. 2, the D23N- and E22G-Aβ40 aggregates showed significant monomer release already at the first datapoint. Notably, in case of wild-type Aβ40, the HSQC spectrum of released monomers was highly similar with that of Aβ40 monomers reported at the same temperature and pressure of 278 K and 2000 bar.[1] The NMR signal intensities were normalized with respect to NMR signal intensities of the same Aβ40 samples measured before aggregation. Since the backbone ^{15}N,^{1}H correlation peaks of Aβ40 followed similar trends of intensity gain during the period of pressure application, the monomer release curves were analyzed through global fitting of the time-dependent intensity (I) data to a mono-exponential equation:

$$I = I_\infty + (I_0 - I_\infty)e^{-t/T} \quad \text{(eq. S1)}$$

where I_0 and I_∞ were residue-specific normalized initial and final intensities and T was a global parameter representing the characteristic time of Aβ40 monomer release. Assuming a minimal two-state model:

$$A \rightleftharpoons M$$

for the conversion between NMR-invisible aggregate (A) and monomeric (M) state where the forward dissociation and backward re-association of monomeric peptide follow first-order kinetics governed by rate constants k_{off} and k_{on}, the fitting parameters obtained from equation (1) were interpreted as:

$$I_\infty = \frac{k_{off}}{k_{off}+k_{on}} \quad \text{(eq. S2)}$$

$$\frac{1}{T} = k_{off} + k_{on} \quad \text{(eq. S3)}$$

MD simulation

In order to simulate the structural and dynamical effects of pressure rise on FAD variants of Aβ40 fibrils, molecular dynamic (MD) simulation method was applied. All MD simulations were carried out with the GROMACS simulation package version 4.6.5 using the Amber99SB-ildn force field parameters as implemented in GROMACS.[2-3] The 2m4j PDB model for the wild-type Aβ40 fibrils,[4] the 2mpz PDB model for the D23N-Aβ40 fibrils,[5] and the 2mvx PDB model for the ΔE22-Aβ40 fibrils [6] were considered as initial structures of MD simulation. The protonation state of the titratable groups were set for neutral pH. For each proposed model, two sets of MD simulation were performed at 300 K and either 1 or 2000 bar. Each fibril model was centered in a sufficiently large periodic box and solvated with pure water using TIP4P-Ew water model.[7] The size of simulation box was 17nm × 17nm × 4.5nm for the wild-type Aβ40, 12.5nm × 12.5nm × 6.5nm for the D23N-Aβ40 and 9nm × 6.5nm × 5nm for the ΔE22-Aβ40. The simulation boxes contained 39400, 28700 and 5800 water molecules, respectively. The total charges of each box were neutralized by replacing water molecules with the required number of sodium and chloride ions. The solvated systems were energy minimized according to the steepest descent algorithm to eliminate bad atomic clashes until the maximum force on atoms reached below 1000 kJ mol^{-1} nm^{-1}. After the energy minimization step in order to set atomic velocities and to adjust pressure and density, each system was equilibrated by applying two successive short-length position-restrained simulations (i.e., 100 ps under a constant volume and temperature and 1 ns under a constant pressure and temperature). For this purpose, temperature was kept at 300 K using the velocity-rescale thermostat,[8] and the pressure was maintained at either 1 or 2000 bar with the Parrinello−Rahman barostat.[9] The LINCS algorithm was utilized to constrain all atomic bonds.[10] A single-range cutoff distance, 1.2 nm, for both Lennard−Jones and short-range electrostatic interactions was applied. The Coulomb interactions for longer distances were modulated by the

Particle Mesh Ewald algorithm.[11] A time step of 2 fs was used for the integration of Newton's equation of motion, and frames were sampled every 2 ps. The duration of each MD simulation was 200 ns. The root mean square fluctuation (RMSF) for backbone atoms of each residue and ionic distances between corresponding functional groups were calculated for trajectories. The collective and correlated motions were detected through the analysis of MD trajectories via principal component analysis (PCA)/essential dynamic (ED) method,[12] which is a useful method to compare the dynamical behavior of similar systems. The PCA method is based on the construction of the covariance matrix of the coordinate fluctuations of CA atoms of peptides along the trajectory after fitting to a suitable reference structure. After diagonalization of the covariance matrix, the information about the concerted motion of proteins could be obtained from a few chosen eigenvectors and eigenvalues that account for the largest variations in the system. In order to investigate the binding free energy of a layer of Aβ40 molecules in fibrillar structure, the MM-PBSA (Molecular Mechanics−Poisson−Boltzmann surface area) method as implemented in g_mmpbsa package was applied.[13] As such, a layer of Aβ40 molecules sandwiched between neighboring layers taken as the ligand and the rest of fibril was set as the binding partner and the MM-PBSA calculations was performed on every 1 ns snapshots of the last 100 ns trajectories. The calculations were performed for different values of dielectric constants (ε of 2, 4, 5, 6 and 7). To compare the results of proposed models, the average binding free energies were normalized for a single chain. The electrostatic surface potential of the cross section area of each fibrillar structures was calculated according to APBS (Adaptive Poisson-Boltzmann Solver) method which solves the equations of continuum electrostatic.[3] Packing efficiencies were calculated as the ratio of van der Waals volume to the Voronoi volume for the initial structures or over the last 100 ns of MD trajectories using the *trjVoronoi* tool.[14]

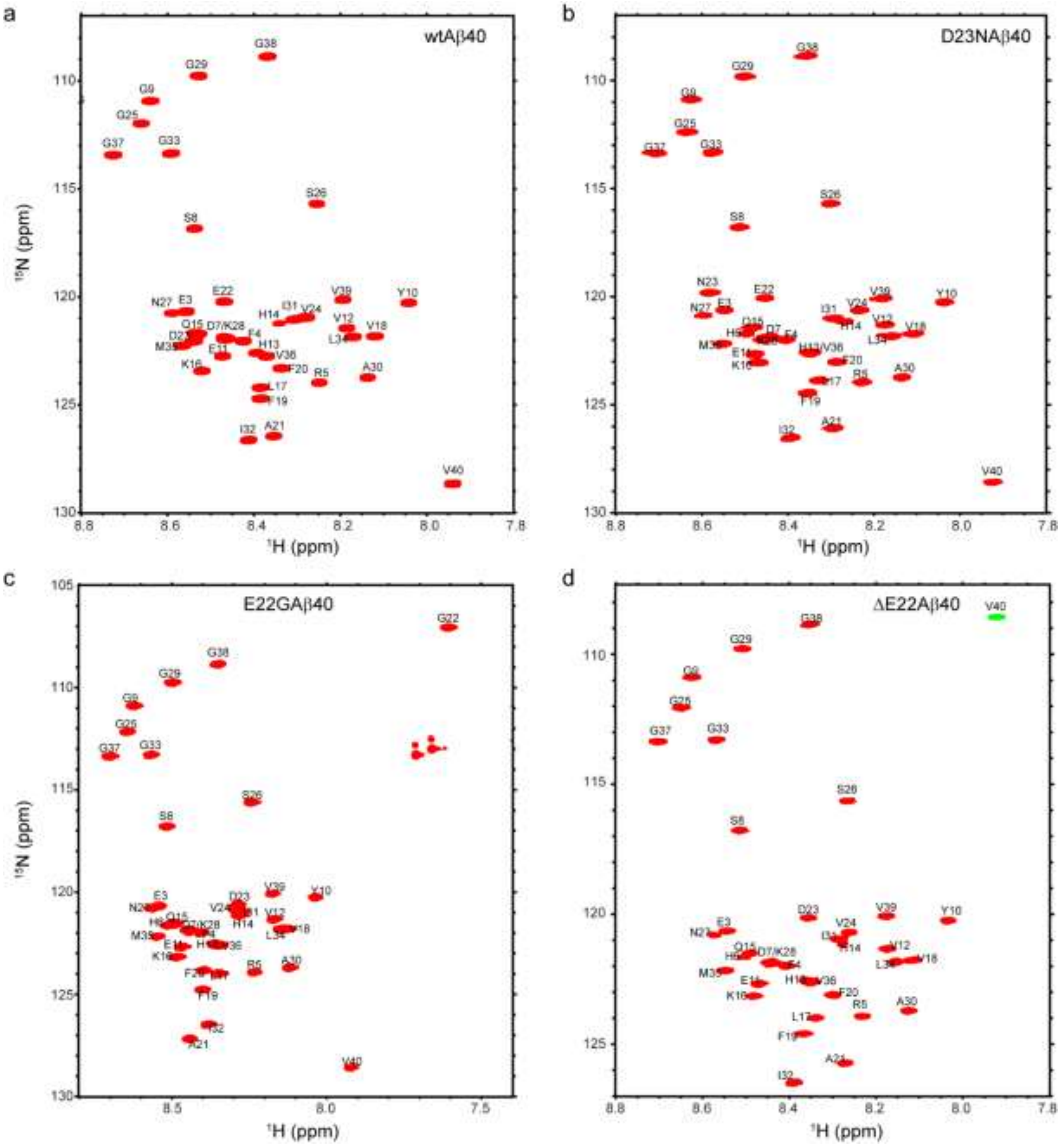

Figure S1. 2D ^{15}N,^{1}H HSQC spectra of Aβ40 variants, i.e. wild-type (a), D23N (b), E22G (c) and ΔE22 (d) Aβ40, measured at 278 K. The peak assignments are shown next to them. In (d), the V40 peak is folded back in the indirect ^{15}N dimension and therefore has an opposite sign compared to other peaks.

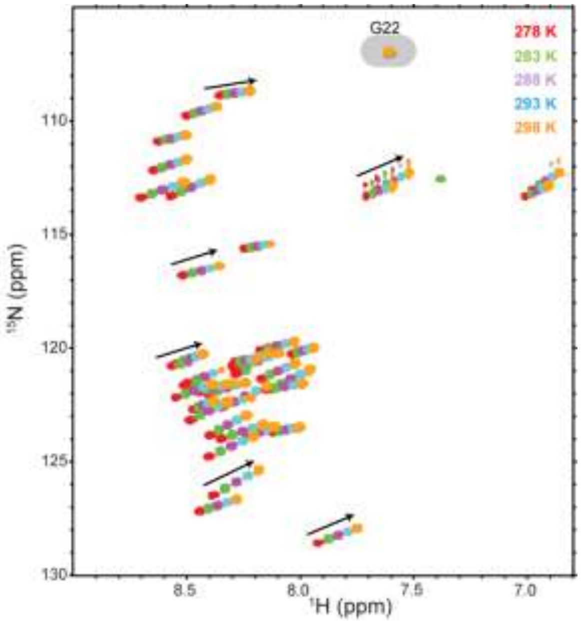

Figure S2. Temperature-dependence of 2D ^{15}N,^{1}H HSQC spectra of Aβ40 variants, shown here as an example for the E22G-Aβ40 peptide. Arrows show the direction of temperature-dependent changes. Note the remarkably up-field HN chemical shift of G22 (shaded area).

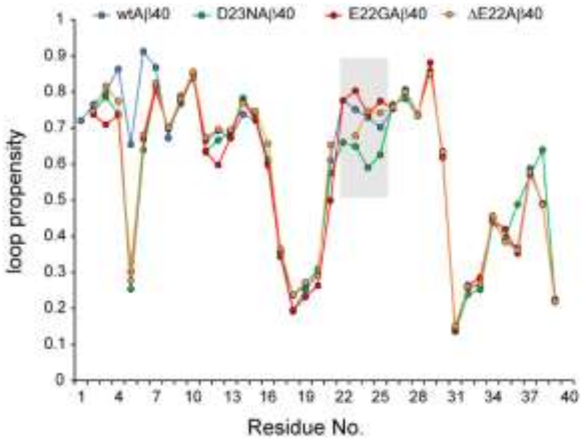

Figure S3. The loop propensity of wild-type, D23N-, E22G- and ΔE22-Aβ40, calculated on the basis of their backbone (CO, Cα, N, HN, Hα) plus Cβ chemical shifts. The shaded area shows how the respective mutations alter the loop propensity around the site of mutation (see also Fig. 3b).

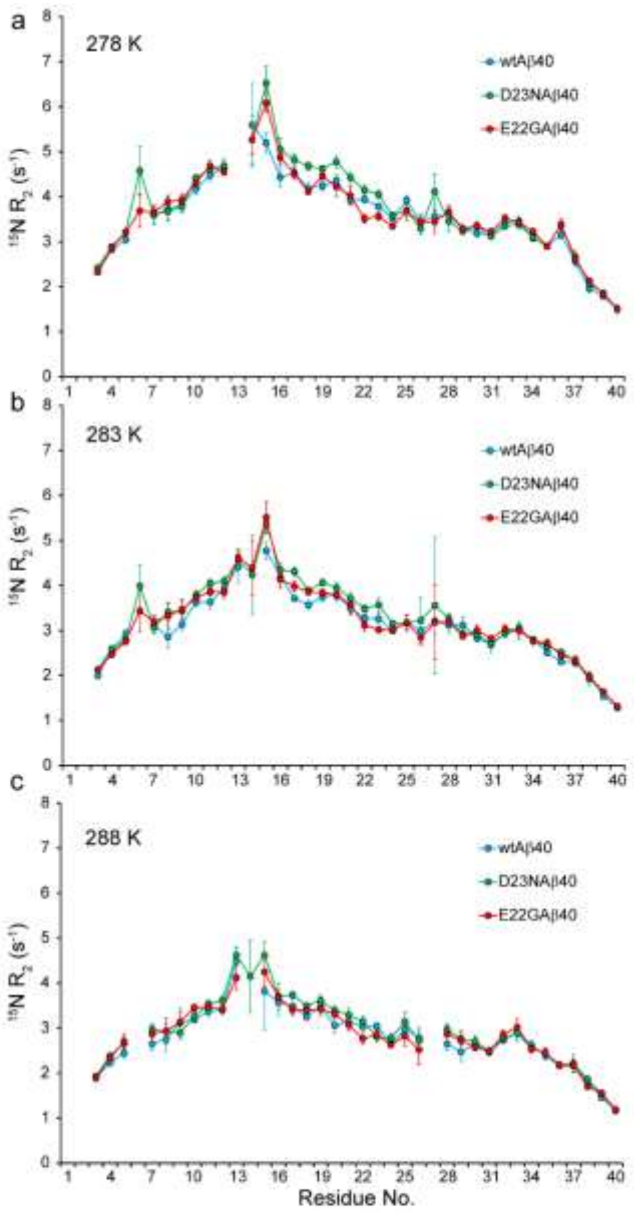

Figure S4. Residue-specific ^{15}N transverse relaxation (R_2) rates of wild-type, D23N- and E22G-Aβ40, measured at 278 (a), 283 (b) and 288 K (c). Upon heating, the R_2 rates of all Aβ40 peptides are reduced, indicating their larger dynamics at pico-to-nanosecond timescale. At 288 K (c), the three Aβ40 peptides exhibit very similar R_2 rates.

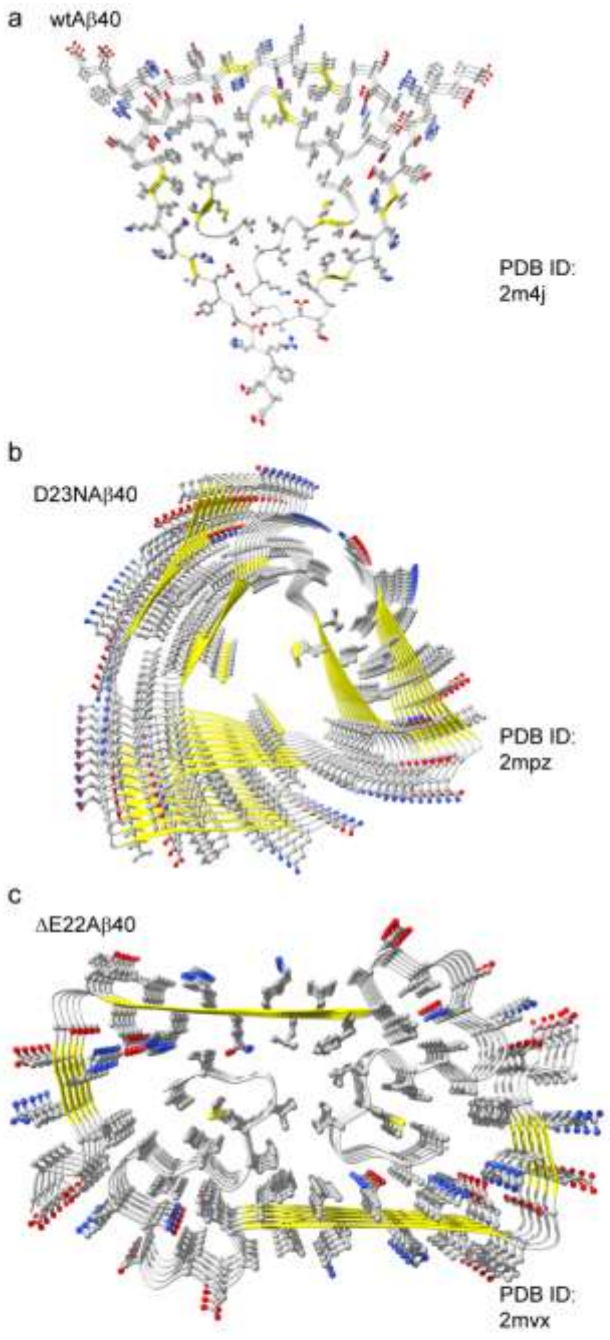

Figure S5. The structure of wild-type, D23N- and ΔE22-Aβ40 fibrils, used for molecular dynamic (MD) simulation. Note the three-fold symmetry in the wild-type (a) and D23N (b) fibrils and the two-fold symmetry in the ΔE22 (c) fibrils about the long axis.

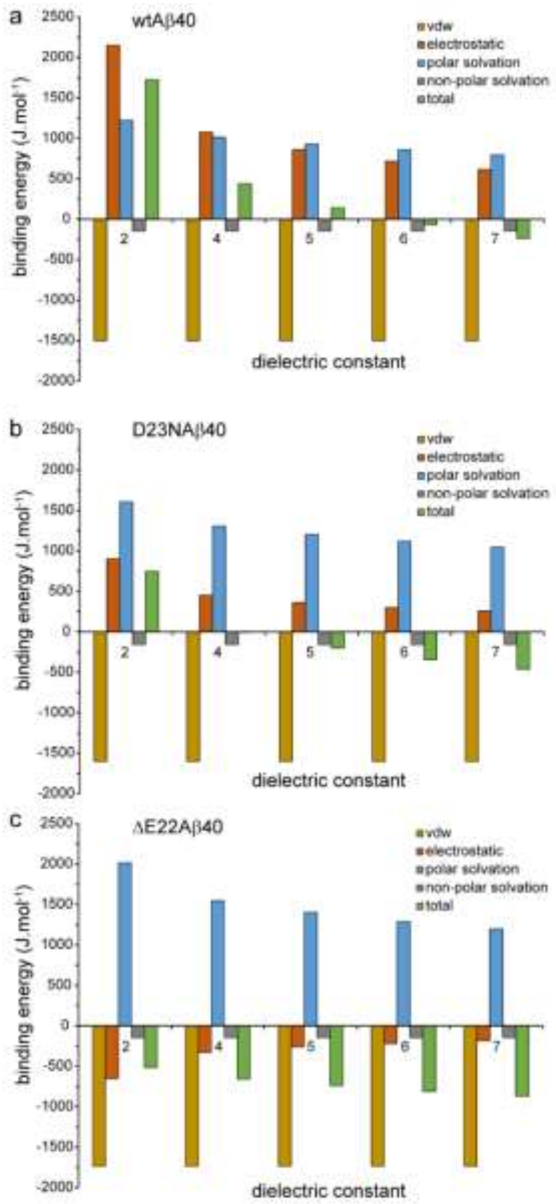

Figure S6. MD-based binding energy per strand as a function of dielectric constant, calculated for the wt- (a), D23N- (b) and ΔE22-Aβ40 (c) fibrils. Different contributions to binding energy, i.e. van der Waals (vdw) and electrostatic interactions and polar and non-polar solvation, are shown. Because of repulsive electrostatic interaction, the total binding energy of wild-type (wt-) and D23N-Aβ40 fibrils become negative only at higher dielectric constants. Conversely, the ΔE22-Aβ40 fibril exhibits attractive electrostatic interaction and negative total binding energy across the studied dielectric constant range.

Table S1. The distance between oppositely charged groups of wild-type, D23N and ΔE22 Aβ40 fibrils, calculated from MD trajectories simulated at two pressure levels of 1 and 2000 bar. The distance ratios between these two pressure levels are reported separately for three classes of pairs of charged groups: intrachain, interchain and interlayer, interchain and intralayer.

	wild-type Aβ40 fibril			D23N- Aβ40 fibril			ΔE22- Aβ40 fibril		
	distance at 1 bar (nm)	distance at 2000 bar (nm)	distance ratio 2000 bar:1 bar	distance at 1 bar (nm)	distance at 2000 bar (nm)	distance ratio 2000 bar:1 bar	distance at 1 bar (nm)	distance at 2000 bar (nm)	distance ratio 2000 bar:1 bar
Intrachain:									
Nterm-E3	0.83	0.88	1.07	0.90	0.92	1.03	0.58	0.62	1.08
E3-R5	1.18	1.28	1.08	0.88	0.85	0.97	1.26	1.24	0.99
R5-D7	0.70	0.73	1.04	0.84	0.85	1.02	0.47	0.54	1.13
D23-K28	0.62	0.56	0.89	-	-	-	-	-	-
Average*			1.02±0.08			1.00±0.03			1.07±0.07
Interchain: Interlayer									
Nterm-E3	1.33	1.14	0.85	1.43	1.52	1.06	0.75	0.80	1.05
D1-K28	-	-	-	-	-	-	1.15	1.18	1.02
E3-R5	1.40	1.36	0.98	1.31	1.29	0.99	1.35	1.34	0.99
E3-K28	-	-	-	-	-	-	0.83	0.81	0.97
R5-D7	0.94	0.86	0.92	1.18	1.22	1.04	0.69	0.72	1.05
D23-K28	0.81	0.75	0.92	-	-	-	-	-	-
R5-E22	1.67	1.32	0.79	-	-	-	-	-	-
E11-K28	-	-	-	0.97	1.00	1.02	-	-	-
Average*			0.89±0.07			1.03±0.03			1.02±0.04
Interchain: Intralayer									
D1-K28	-	-	-	-	-	-	0.94	1.01	1.08
R5-E22	1.64	1.20	0.73	-	-	-	0.51	0.48	0.96
E11-K28	-	-	-	0.82	0.80				
Average*			0.73			0.97			1.02
Average**			0.93±0.12			1.01±0.03			1.03±0.06

*. Mean±SD of distance ratios calculated separately for the intrachain, interchain-interlayer and interchain-intralayer distances. **. Mean±SD of all the calculated distance ratios.

Supplementary references

(1) Vemulapalli, S. P. B.; Becker, S.; Griesinger, C.; Rezaei-Ghaleh, N. Combined high-pressure and multiquantum NMR and molecular simulation propose a role for N-terminal salt dridges in amyloid-beta. *J. Phys. Chem. Lett.* **2021,** *12*, 9933-9939.

(2) Berendsen, H. J. C.; Vanderspoel, D.; Vandrunen, R. Gromacs - a message-passing parallel molecular-dynamics implementation. *Comput. Phys. Commun.* **1995,** *91*, 43-56.

(3) Hornak, V.; Abel, R.; Okur, A.; Strockbine, B.; Roitberg, A.; Simmerling, C. Comparison of multiple Amber force fields and development of improved protein backbone parameters. *Proteins* **2006,** *65*, 712-725.

(4) Lu, J. X.; Qiang, W.; Yau, W. M.; Schwieters, C. D.; Meredith, S. C.; Tycko, R. Molecular structure of beta-amyloid fibrils in Alzheimer's disease brain tissue. *Cell* **2013,** *154*, 1257-1268.

(5) Sgourakis, N. G.; Yau, W. M.; Qiang, W. Modeling an In-register, parallel "Iowa" A beta fibril structure using solid-state NMR data from labeled samples with Rosetta. *Structure* **2015,** *23*, 216-227.

(6) Schutz, A. K.; Vagt, T.; Huber, M.; Ovchinnikova, O. Y.; Cadalbert, R.; Wall, J.; Guntert, P.; Bockmann, A.; Glockshuber, R.; Meier, B. H. Atomic-resolution three-dimensional structure of amyloid beta fibrils bearing the Osaka mutation. *Angew. Chem. Int. Ed. Engl.* **2015,** *54*, 331-335.

(7) Jorgensen, W. L.; Chandrasekhar, J.; Madura, J. D.; Impey, R. W.; Klein, M. L. Comparison of simple potential functions for simulating liquid water. *J. Chem. Phys.* **1983,** *79*, 926-935.

(8) Berendsen, H. J. C.; Postma, J. P. M.; Vangunsteren, W. F.; Dinola, A.; Haak, J. R. Molecular-dynamics with coupling to an external bath. *J. Chem. Phys.* **1984,** *81*, 3684-3690.

(9) Berendsen, H. J. C.; Grigera, J. R.; Straatsma, T. P. The missing term in effective pair potentials. *J. Phys. Chem.* **1987,** *91*, 6269-6271.

(10) Hess, B. B., H.; Berendsen, H.J.C.; Fraaije, J.G.E.M. LINCS: A linear constraint solver for molecular simulations. *J. Comput. Chem.* **1998,** *18*, 1463-1472.

(11) Darden, T.; York, D.; Pedersen, L. Particle Mesh Ewald - an N.Log(N) method for Ewald sums in large systems. *J. Chem. Phys.* **1993,** *98*, 10089-10092.

(12) Amadei, A.; Linssen, A. B. M.; Berendsen, H. J. C. Essential dynamics of proteins. *Proteins* **1993,** *17*, 412-425.

(13) Kumari, R.; Kumar, R.; Open Source Drug Discovery, C.; Lynn, A. g_mmpbsa--a GROMACS tool for high-throughput MM-PBSA calculations. *J. Chem. Inf. Model* **2014,** *54*, 1951-1962.

(14) Abel, S.; Dupradeau, F. Y.; Marchi, M. Molecular dynamics simulations of a characteristic DPC micelle in water. *J. Chem. Theory Comput.* **2012,** *8*, 4610-4623.
